# Inactivation of Spoilage Yeasts by *Mentha spicata* L. and *M*. × *villosa* Huds. Essential Oils in Cashew, Guava, Mango, and Pineapple Juices

**DOI:** 10.3389/fmicb.2018.01111

**Published:** 2018-05-25

**Authors:** Erika T. da Cruz Almeida, Isabella de Medeiros Barbosa, Josean F. Tavares, José M. Barbosa-Filho, Marciane Magnani, Evandro L. de Souza

**Affiliations:** ^1^Laboratorio de Microbiologia de Alimentos, Departamento de Nutrição, Centro de Ciências da Saúde, Universidade Federal da Paraíba, João Pessoa, Brazil; ^2^Núcleo de Caracterização e Análises, Instituto de Pesquisa em Medicamentos, Universidade Federal da Paraíba, João Pessoa, Brazil; ^3^Laboratorio de Processos Microbianos em Alimentos, Departamento de Engenharia de Alimentos, Centro de Tecnologia, Universidade Federal da Paraíba, João Pessoa, Brazil

**Keywords:** *Mentha* spp., essential oil, anti-yeast effects, fruit juice, preservation

## Abstract

This study evaluated the efficacy of the essential oil from *Mentha spicata* L. (MSEO) and *M*. × *villosa* Huds. (MVEO) to inactivate *Candida albicans, C. tropicalis, Pichia anomala* and *Saccharomyces cerevisiae* in Sabouraud dextrose broth and cashew, guava, mango, and pineapple juices during 72 h of refrigerated storage. The effects of the incorporation of an anti-yeast effective dose of MSEO on some physicochemical and sensory characteristics of juices were evaluated. The incorporation of 3.75 μL/mL MSEO or 15 μL/mL MVEO caused a ≥5-log reductions in counts of *C. albicans, P. anomala*, and *S. cerevisiae* in Sabouraud dextrose broth. In cashew and guava juices, 1.875 μL/mL MSEO or 15 μL/mL MVEO caused ≥5-log reductions in counts of *P. anomala* and *S. cerevisiae*. In pineapple juice, 3.75 μL/mL MSEO caused ≥5-log reductions in counts of *P. anomala* and *S. cerevisiae*; 15 μL/mL MVEO caused ≥5-log reductions in counts of *S. cerevisiae* in mango juice. The incorporation of 1.875 μL/mL MSEO did not affect the physicochemical parameters of juices and did not induce negative impacts to cause their possible sensory rejection. These results show the potential of MSEO and MVEO, primarily MSEO, to comprise strategies to control spoilage yeasts in fruit juices.

## Introduction

In last years, there is an increasing search by consumers for foods possessing particular characteristics in their composition (e.g., low contents of simple sugars, sodium, fat, and cholesterol) and the presence of constituents with health promoting effects (e.g., polyphenols and other antioxidants) (Corbo et al., 2014). Consumers have also demanded for fresher foods submitted to minimal processing having low or no amounts of synthetic preservatives (Román et al., 2017). In this context, the consumption and variety of unpasteurized and synthetic additive-free fruit juices in market has been increased. Nonetheless, the non-use of heating treatments and synthetic preservatives facilitate the survival of microbial contaminants in fruit juices resulting in decreased stability and higher risk to consumers (Leite et al., 2016).

In contrast to the harmful effects on some potentially pathogenic bacteria, the low pH and high sugar content in fruit juices provide a favorable substrate to yeast growth (Tribst et al., 2009). Most of the yeast species are highly fermentative and metabolize sugars with the production of alcohols (mostly ethanol), carbon dioxide and other organic compounds causing packing blown and unpleasing taste and flavor in fruit juices. Additionally, yeast growth in fruit juice may result in increased turbidity, flocculation, pellicle formation, and clumping (Lawlor et al., 2009). Reports have cited *Candida, Pichia, Rhodotorula*, and *Saccharomyces* as yeast genera frequently involved in fruit juice contamination and spoilage (Tournas et al., 2006; Vantarakis et al., 2011; Aneja et al., 2014). Although the fruit juice yeast contamination has not been associated with risks of foodborne diseases, the yeast growth in these products may result in alterations that cause consumption rejection, decreased market value and economic loss to processors (Tournas et al., 2006; Lawlor et al., 2009).

Control of contaminant yeasts in fruit juices has been classically achieved by the application of heat and/or incorporation of synthetics preservatives (Amirpour et al., 2015). Pasteurization is the method more commonly used to control microorganism in fruit juices, but the heating temperature used in this procedure may impact negatively on their physicochemical and sensory characteristics (Sánchez et al., 2017). Additionally, synthetic preservatives more commonly used in fruit juices (e.g., benzoic acid, sorbic acid and sulfur dioxide) may have harmful effects to consumers such as allergies, asthma, and skin rashes (Vally et al., 2009). Efforts have been addressed to the study of non-synthetic antimicrobials to inactivate contaminant microorganisms in fruit juices (Chanukya and Rastogi, 2017).

Interest in using non-synthetic antimicrobials has increased the attention to essential oils as potential inhibitors of microorganisms to preserve fruit juices. Essential oils are categorized as “generally recognized as safe” (GRAS) for use as flavoring ingredients in beverages (U.S. Food and Drug Administration [USFDA], 2015). *Mentha* genus, Lamiaceae family, includes more than 25 plant species that are recognized for their aromatic properties, being exploited in various sectors including pharmacy, cosmetic, agronomy, and foods (Park et al., 2016; Mamadalieva et al., 2017). *Mentha spicata* L., commonly known as spearmint, is one of the most common, popular and extensively studied *Mentha* species. *Mentha* × *villosa* Huds., also reported in literature as *M. crispa* and commonly known as creeping mint, is a hybrid of *M. spicata* and *M. suaveolens*. *M.* × *villosa* is still few explored concerning its biological activities, but has been considered a potential alternative for use as flavoring agent in foods and beverages (Lahlou et al., 2002; Kumar et al., 2011).

Essential oils from *Mentha* species have shown effective to inhibit a variety of pathogenic bacteria and molds (Guerra et al., 2015; Leite et al., 2016; Park et al., 2016; de Sousa Guedes et al., 2016) but few studies have focused on their anti-yeast effects. Previous studies have reported the anti-yeast effects of *M. piperita* essential oil (Tyagi et al., 2013; Ferreira et al., 2014; Karaman et al., 2016); however, there is a lack of studies about the efficacy of the essential oil from *M. spicata* (MSEO) and *M. villosa* (MVEO) to inactivate spoilage yeasts in juices.

Cashew, guava, mango, and pineapple juice are some of the main fruit species cultivated in Brazil (Food and Drug Administration [FAO], 2015), with much of their production destinated for juice processing. A variety of well-accepted fruit products (such as frozen pulps, unpasteurized juices, ready-to-eat fruit-mix salads and minimally processed slices) containing grinded mint (*Mentha* spp.) leaves are available in the market, reinforcing the idea that fruit juices could represent potential matrices to exploit the antimicrobial properties of MSEO and MVEO. This study evaluated the efficacy of MSEO and MVEO to inactive the potentially spoilage yeasts *C. albicans, C. tropicalis, P. anomala*, and *S. cerevisiae* in pineapple, cashew, guava, and mango juice stored under refrigeration. The effects of the incorporation of an effective anti-yeast dose of MSEO on some physicochemical parameters and sensory characteristics of theses juices were assessed.

## Materials and Methods

### Test Strains and Growth Conditions

Different yeasts species commonly cited as potential spoilage agents of fruit juices (Tournas et al., 2006; Vantarakis et al., 2011; Aneja et al., 2014) were used as test organisms, to cite: *C. albicans* (type-strain ATCC 90028), *C. tropicalis* (type-strain ATCC 28707), *P. anomala* (type-strain ATCC 40101) and *S. cerevisiae* (type-strain ATCC 2601). These type-strains were supplied as freeze-dried isolates by the Collection of Reference Microorganisms, National Institute of Quality Control in Health, Oswaldo Cruz Foundation (Rio de Janeiro, Brazil), and they were activated by two consecutive 48 h-passages in Sabouraud dextrose broth (Acumedia Manufacturers Inc., Lansing, MI, United States) at 30°C. Stocks were kept in Sabouraud dextrose broth containing glycerol (15 g/100 mL) at -20°C; working cultures were maintained in Sabouraud dextrose agar (Acumedia Manufacturers Inc., Lansing, MI, United States) at 4°C, and transferred for fresh Sabouraud dextrose agar monthly. For use in assays, the strains were first cultivated in Sabouraud dextrose agar at 30°C during 48 h, harvested by centrifugation (4500 × *g* × 15 min, 4°C), washed twice in sterile saline solution (0.85 g/100 mL) and re-suspended in sterile saline solution to obtain cell suspensions presenting optical density reading at 625 nm of 0.75 for *C. albicans, C. tropicalis*, and *P. anomala*, and of 0.95 for *S. cerevisiae*. These suspensions provided viable counts of approximately 7 log cfu/mL when pour plated onto Sabouraud dextrose agar.

### Preparation of Fruit Juices

Cashew (*Anacardium occidentale* L.), guava (*Psidium guajava* L.), mango (*Mangifera indica* L.), and pineapple (*Ananas comosus* L. Merrill) fruit were acquired from a local wholesale distributor (João Pessoa, Brazil) and selected in commercial maturation stage, with absence of mechanical damages and visible signs of infection. Fruit were surface disinfected through immersion in a sodium hypochlorite solution (150 ppm, pH 7.2 adjusted using 1 M NaOH) for 5 min, washed with distilled water and dried for 30 min in a biosafety cabinet. Fruit were aseptically peeled, chopped and mixed with sterile distilled water (50 g of fruit in 100 mL of sterile distilled water for guava juice and 60 g of fruit in 100 mL of sterile distilled water for cashew, mango, and pineapple; Anonymous, 2003) using a domestic blender (for 3 min). The fruit juices were double filtered using a triple-cheesecloth layer and sterilized using Wattman membrane filters nylon por size 0.22 μm (Sigma-Aldrich, St. Louis, MO, United States). Juices samples were stored in 25-mL aliquots at -20°C, and, when necessary, aliquots were thawed under refrigeration (4 ± 0.5°C) and used in assays.

### MSEO and MVEO

MSEO and MVEO extracted through steam distillation were obtained from Ferquima Ind. Com. Ltd. (São Paulo, Brazil) and Hebron^®^ company (Recife, Brazil), respectively. A stock emulsion of each essential oil at a concentration of 240 μL/mL was prepared in sterilized Sabouraud dextrose broth containing Tween 80 (1%, v/v; Sigma-Aldrich, St. Louis, MO, United States) as an emulsifier (de Sousa Guedes et al., 2016). At the highest assayed concentration (1%, v/v), Tween 80 presented no inhibitory effect against the tested yeast strains. Stock emulsions of each essential oil were maintained in an amber screw-capped tube under 4 ± 0.5°C and used in anti-yeast assays after a storage time period no longer than 48 h.

### Identification of Essential Oils Constituents

MSEO and MVEO constituents were identified using a gas chromatograph coupled to mass spectrometer (CGMS-QP2010 Ultra Shimadzu, Kyoto, Japan). Analytical conditions were: a RTX-5MS capillary column (30 m × 0.25 mm × 0.25 μm); program temperature: 60–240°C (3°C/min); injector temperature: 250°C; detector temperature: 220°C; carrier gas: helium adjusted to 0.99 mL/min; ionizing energy: 70 eV; and mass range: 40–500. Identification of each component was performed by comparing their mass spectra with the NIST/EPA/NIH Mass Spectral Database (National Institute of Standards Technology, Norwalk, CT, United States) and FFNSC1.3 (Flavor and Fragrance Natural and Synthetic Compounds) libraries as well as the Kovats retention index (Adams, 2001). Quantification of the essential oils constituents was obtained after normalizing the areas of each detected constituent and expressed as a percentage of area (%) (de Sousa Guedes et al., 2016).

### Determination of the Minimum Inhibitory Concentration of Essential Oils

MIC of MSEO and MVEO was determined using a microdilution in broth assay (Clinical and Laboratory Standards Institute [CLSI], 2008). For this, 50 μL-aliquots of the stock emulsion of MSEO or MVEO (240 μL/mL) were dispensed into wells of a 96-well microplate containing 50 μL of double concentrate Sabouraud dextrose broth. Subsequently, 50 μL-aliquots were transferred to the following wells, and through geometric dilutions the essential oils concentrations varied from 120 to 0.469 μL/mL. Afterward, 50 μL-aliquots of the yeast suspensions were added to each well (final viable counts were approximately 7 log cfu/mL), and the final essential oils concentrations varied from 60 to 0.234 μL/mL. Each microplate contained a positive (Sabouraud dextrose broth inoculated) and a negative (Sabouraud dextrose broth non-inoculated) control for each yeast strain tested. The microplate was loosely wrapped with cling wrap to avoid essential oil volatilization, and incubated at 30°C for 48 h. MIC was determined as the lowest concentration of each essential oil required to prevent visible yeast growth.

### Effects of the Essential Oils on Yeasts Counts in Sabouraud Dextrose Broth and Fruit Juices Over 72 h

The effects of different concentrations of MSEO and MVEO on the yeasts counts were evaluated in Sabouraud dextrose broth and in cashew, guava, mango, and pineapple juice during 72 h of refrigerated storage (4 ± 0.5°C) using a viable cell count method (de Souza et al., 2007). Initially, 2 mL-aliquots of Sabouraud dextrose broth or fruit juice samples containing MSEO or MVEO in an amount enough to provide the required final concentrations of 3.75, 1.875, and 0.937 μL/mL or 15, 7.5 and 3.75 μL/mL, respectively, were inoculated with 2 mL of the yeast suspension and vortexed for 30 s. The mixtures were maintained at 4 ± 0.5°C, and at different storage time intervals (0 – just after homogenization, 5, 15, 30, 45 min and 1, 2, 4, 8, 12, 24, 48, and 72 h), a 100 μL-aliquot of each mixture was serially diluted in sterilized saline solution (0.85 g/100 mL, w/v), and 10 μL-aliquots of each dilution were inoculated onto Sabouraud dextrose agar using a microdrop technique (Herigstad et al., 2001). Additionally, 100 μL-aliquot of each mixture was directly inoculated onto Sabouraud dextrose agar. Inoculated control juices not-containing MSEO or MVEO were assayed similarly. After an incubation period of 48–72 h at 30°C, the visible colonies were counted, and the results were expressed as log cfu/mL. Plates inoculated with aliquots collected from juice samples containing MSEO or MVEO were incubated for an additional 24 h at adequate temperature compared with the samples collected from control juice.

Reduction in yeast counts were calculated using the formula: log N_0_ – log N_;_ where log N_0_ and log N were the initial count and count after incubation, respectively, for indicated storage time interval. Results were expressed as log cycle reduction. The detection limit of the test was 1 log cfu/mL.

### Analysis of Physicochemical Parameters of Fruit Juices

Total soluble solids, pH and titratable acidity values were determined in cashew, guava, mango, and pineapple juice with and without MSEO (1.875 μL/mL) on time zero (baseline, just after the essential oil incorporation and homogenization) and after 72 h of refrigerated storage (4 ± 0.5°C). These parameters were selected because they comprise the current Brazilian physicochemical standards to unsweetened fruit juices (Anonymous, 2003). Soluble solids content (°Brix) was analyzed using a digital refractometer (model HI 96801, Hanna Instruments, São Paulo, Brazil) (Association of Official Analytical Chemists International [AOAC], 2016). pH values were determined using a digital potentiometer (model Q400AS, Quimis, São Paulo, Brazil) (Association of Official Analytical Chemists International [AOAC], 2016). Titratable acidity was determined employing phenolphthalein as indicator with NaOH to the 0.1 N, and the results were expressed in grams per 100 mL of citric acid equivalents (Association of Official Analytical Chemists International [AOAC], 2016).

### Sensory Analysis of Fruit Juices

Sensory analyses were performed after the approval from an Ethics Research Committee (Federal University of Paraía – Brazil) under a protocol number 1.125.993/2015. Sensory evaluation was performed using check-all-that-apply (CATA) questions for cashew, guava, mango, and pineapple juices with 1.875 μL/mL of MSEO. Juices were produced in the same day of the sensory tests and maintained under refrigeration storage (4 ± 0.5°C). Firstly, most mentioned appropriate terms for each attribute (color, appearance, flavor, odor, and texture) of the all fruit juices were established by an untrained group to compose the CATA questions (**Table 1**). One hundred untrained panelists (17–50 years old), for each juice, were recruited and selected considering their habits of consuming fruit juices two or more times per week. Sensory analyses were performed in four testing sessions, one for each juice, conducted in individual cabins with white light. Panelists evaluated the juice samples, immediately after removal from refrigerated storage, served in 30-mL aliquots in white disposable cups in individual booths with controlled temperature and lighting. Panelists were asked to check all the terms of CATA questions (as previously defined) appropriate to describe each attribute (Ares et al., 2010).

**Table 1:** Terms surveyed for check-all-that-apply (CATA) questions of each sensory attribute of the fruit juices evaluated.

Appearance	Flavor	Odor
Characteristic color	Sweet	Characteristic odor
of the juice		of fruit
Uncharacteristic color	Not sweet	Uncharacteristic odor
of the juice	Strange flavor	of fruit
	Refreshing sensation	Mint odor
	Fruit flavor	
	Mint flavor	
	Bitter flavor	
	Pleasant flavor	
	Unpleasant flavor	

### Statistical Analysis

The assays were performed in three independent experiments in triplicate. Different fruit juices batches (prepared using a pool of at least three different fruit) and standardized inoculum from a single yeast suspension prepared from two independent cultures of the test yeast were used in each independent experiment. Results of MIC determination assays are expressed as modal values because the MIC values were the same in all repetitions. For the yeast count assays and physicochemical parameters, the statistical analyses were performed to determine significant differences (*p* ≤ 0.05) based on Student’s *t*-test or ANOVA followed by *post hoc* Tukey test. The computational software Sigma Stat 3.5 software (Jandel Scientific Software, San Jose, CA, United States) was used for these statistical analyses.

Data from CATA questions were analyzed by determining the frequency of use of each term for describing each attribute of the fruit juices. Significant differences (*p* ≤ 0.05) among samples were evaluated using Cochran’s Q Test (Manoukian, 1986). MedCalc Statistical Software version 18 (MedCalc Software bvba, Ostend, Belgium) was used for this analysis.

## Results and Discussion

### Identification of MSEO and MVEO Constituents

Constituents identified in MSEO and MVEO are shown in **Table 2**. Majority constituents in MSEO were carvone (72.69%) and limonene (14.25%), other constituents were identified in lower amounts, e.g., menthol (2.29%) and menthone (1.07%). Piperitenone oxide (62.39%), eucalyptol (4.46%), and limonene (4.40%) were the majority constituents in MVEO, followed by other constituents in lower amounts, such as isopentyloxyethyl acetate (2.49%), β-pinene (2.14%), myrcene (2.01%), α-pinene (1.52%), sabinene (1.41%), β-caryophyllene (1.34%), 6-methyl-5-hepten-2-ol (1.31%), piperitone oxide (1.25%), germacrene D (1.23%) and *p*-cymene (1.04%). A wide variety of other constituents were identified in amounts < 1% in both MSEO and MVEO.

**Table 2:** Constituents identified in the essential oil from *Mentha spicata* L. (MSEO) and *M.* × *villosa* Huds. (MVEO) in amounts > 1%.

MSEO				MVEO			
Retention time	Kovats index	%	Constituent	Retention time	Kovats index	%	Constituent
8.824	1027	14.25	Limonene	5.893	932	1.52	α-Pinene
13.805	1152	1.07	Menthone	7.002	972	1.41	Sabinene
14.638	1171	2.29	Menthol	7.129	976	2.14	β-Pinene
18.019	1248	72.69	Carvone	7.478	988	2.01	Myrcene
				7.605	993	1.31	6-Methyl-5-hepten-2-ol
				8.661	1023	1.04	*p*-Cymene
				8.814	1027	4.40	Limonene
				8.914	1029	4.46	Eucalyptol
				14.857	1177	2.49	Isopentyloxyethyl acetate
				18.295	1245	1.25	Piperitone oxide
				23.468	1371	62.39	Piperitenone-oxide
				25.564	1420	1.34	β-Caryophyllene
				28.183	1482	1.23	D-Germacrene

Existence of different chemotypes based on qualitative and quantitative differences within essential oils from the same plant species is a common characteristic in *Mentha* genus (Edris et al., 2003). Nine different chemotypes has been already reported to *Mentha* species (Kokkini and Vokou, 1989; Mimica-Dukiæ, 2008). Considering the detected majority constituents, MSEO and MVEO evaluated in this study could be classified as belonging to the chemotypes carvone-dihydrocarvone and piperitenone oxide, respectively.

### Anti-yeast Effects of MSEO and MVEO

MSEO and MVEO displayed MIC values of 1.875 and 7.5 μL/mL, respectively, against *C. albicans, C. tropicalis, P. anomala*, and *S. cerevisiae*. No previous studies evaluating the inhibitory effects of MSEO against *C. tropicalis* and *P. anomala* were found in literature, and the available studies reported MIC of 0.78 μL/mL against *S. cerevisiae* (Liu et al., 2012) and MIC varying from 20 to 40 μL/mL against *C. albicans* (Martins et al., 2012; Bona et al., 2016). Regarding the MVEO, most of the available studies has focused on its antiparasitic activities (de Sousa Guedes et al., 2016; Matos-Rocha et al., 2016). Only a previous study reported the efficacy of neat MVEO to inhibit *C. albicans* with the measure of growth inhibition zones using an agar diffusion assay (Arruda et al., 2006).

Inhibitory effects of MSEO and MVEO toward the test yeast strains could be related to the already reported antifungal properties of their majority compounds. Antifungal effects of carvone involve primarily two different action modes, to cite: (i) partition into the cell membrane, causing inhibition of plasma membrane H+-ATPase and intracellular acidification; and (ii) inhibition of ergosterol biosynthesis pathway, reducing its amounts in cell membranes with disturbance of membrane fluidity and cell integrity (Samber et al., 2015). Piperitone oxide, as an oxygenated monoterpene, may induce disorganization of cell membrane structures, resulting in depolarization and physical or chemical alterations, thereby disrupting fungal metabolic activities (Ait-Ouazzou et al., 2012; Guerra et al., 2015). In addition to the majority constituents, other compounds detected in lower amounts in MSEO and MVEO such as caryophyllene, eucalyptol, myrcene, and pulegone have demonstrated inhibitory effects against a variety of microorganisms (Zengin and Baysal, 2014; Dahham et al., 2015; Guerra et al., 2015).

**FIGURE 1:**
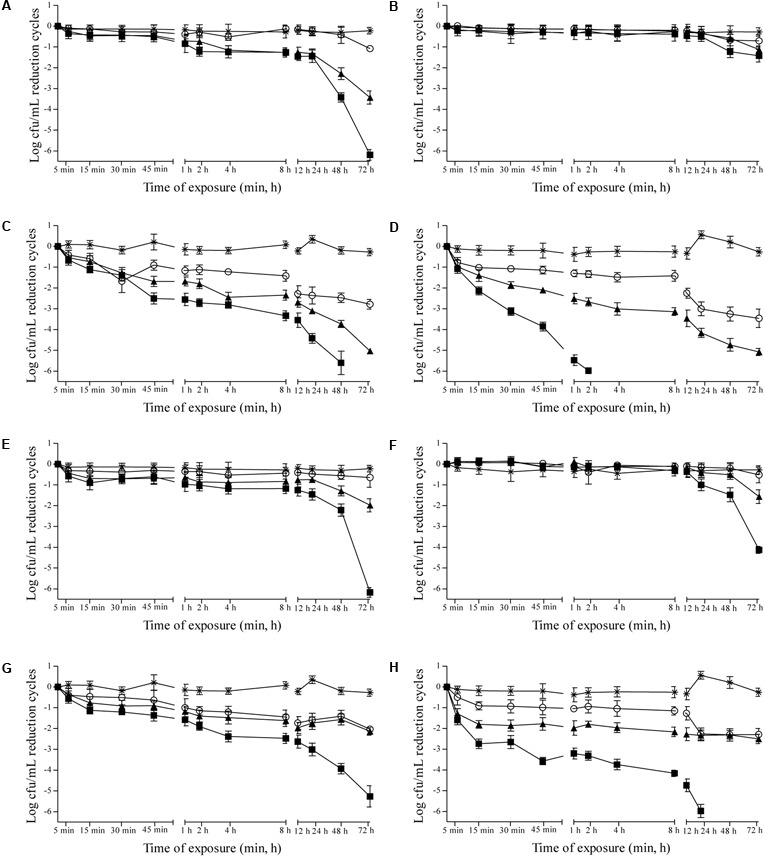
Reduction cycles (log cfu/mL) of the counts of *C. albicans* ATCC 90028 **(A,E)**, *C. tropicalis* ATCC 28707 **(B,F)**, *P. anomala* ATCC 40101 **(C,G)** and *S. cerevisiae* ATCC 2601 **(D,H)** in cashew juice at 4 ± 0.5°C as a function of the concentration of *M. spicata* L. essential oil **(A–D)** at (■): 3.75 μL/mL, (▲): 1.875 μL/mL, (∘): 0.9375 μL/mL or *M. villosa* Huds. essential oil **(E–H)**. (■): 15.0 μL/mL, (▲): 7.5 μL/mL, (∘): 3.75 μL/mL, (^∗^) control: 0 μL/mL. Detection limit of the test: 1 log cfu/mL.

**FIGURE 2:**
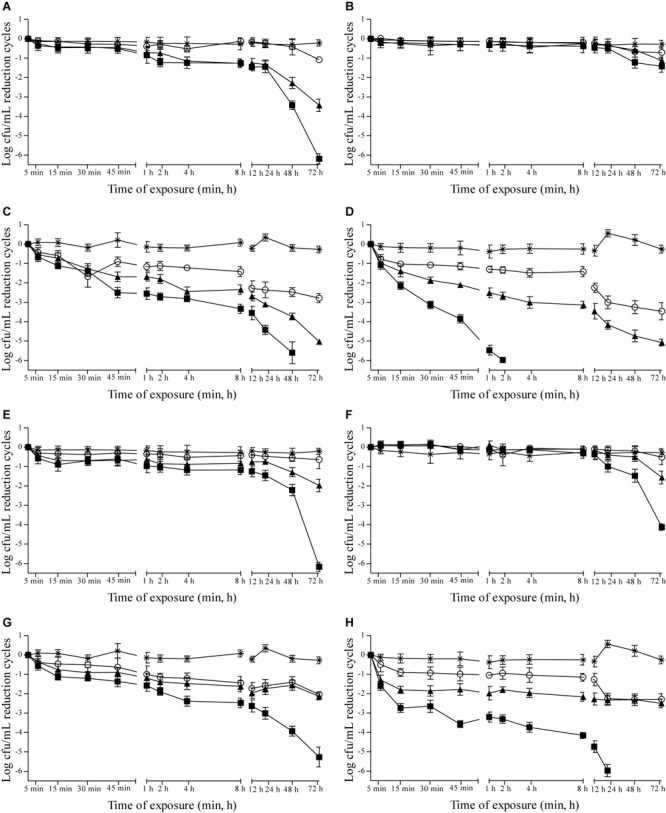
Reduction cycles (log cfu/mL; *n* = 6) itC. albicans ATCC 90028 **(A,E)**, *C. tropicalis* ATCC 28707 **(B,F)**, *P. anomala* ATCC 40101 **(C,G)** and *S. cerevisiae* ATCC 2601 **(D,H)** in guava juice at 4 ± 0.5°C as a function of the concentration of *M. spicata* L. essential oil **(A–D)** at (■): 3.75 μL/mL, (▲): 1.875 μL/mL, (∘): 0.9375 μL/mL or *M. villosa* Huds. essential oil **(E–H)**. (■): 15.0 μL/mL, (▲): 7.5 μL/mL, (∘): 3.75 μL/mL, (^∗^) control: 0 μL/mL. Detection limit of the test: 1 log cfu/mL.

**FIGURE 3:**
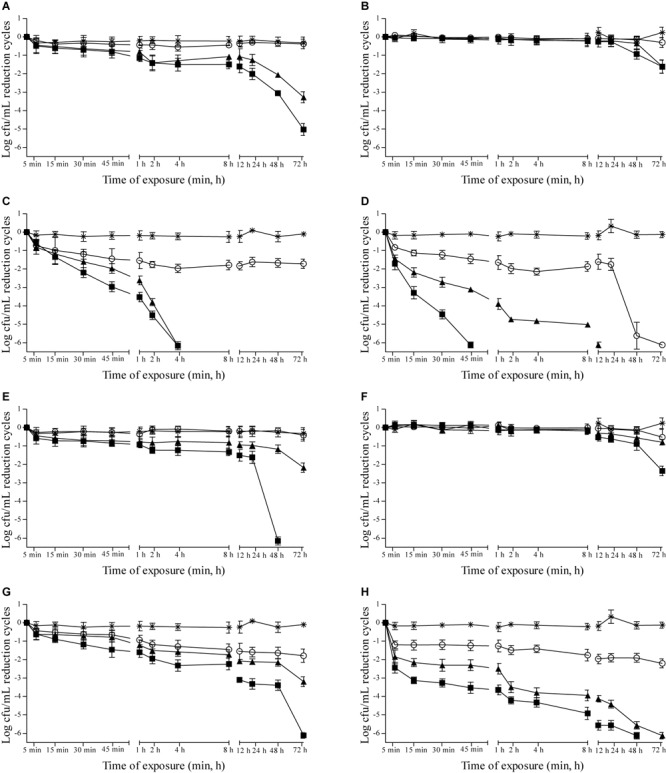
Reduction cycles (log cfu/mL; *n* = 6) of the counts of *C. albicans* ATCC 90028 **(A,E)**, *C. tropicalis* ATCC 28707 **(B,F)**, *P. anomala* ATCC 40101 **(C,G)** and *S. cerevisiae* ATCC 2601 **(D,H)** in mango juice at 4 ± 0.5°C as a function of the concentration of *M. spicata* L. essential oil **(A–D)** at (■): 3.75 μL/mL, (▲): 1.875 μL/mL, (∘): 0.9375 μL/mL or *M. villosa* Huds. essential oil **(E–H)**. (■): 15.0 μL/mL, (▲): 7.5 μL/mL, (∘): 3.75 μL/mL, (^∗^) control: 0 μL/mL. Detection limit of the test: 1 log cfu/mL.

**FIGURE 4:**
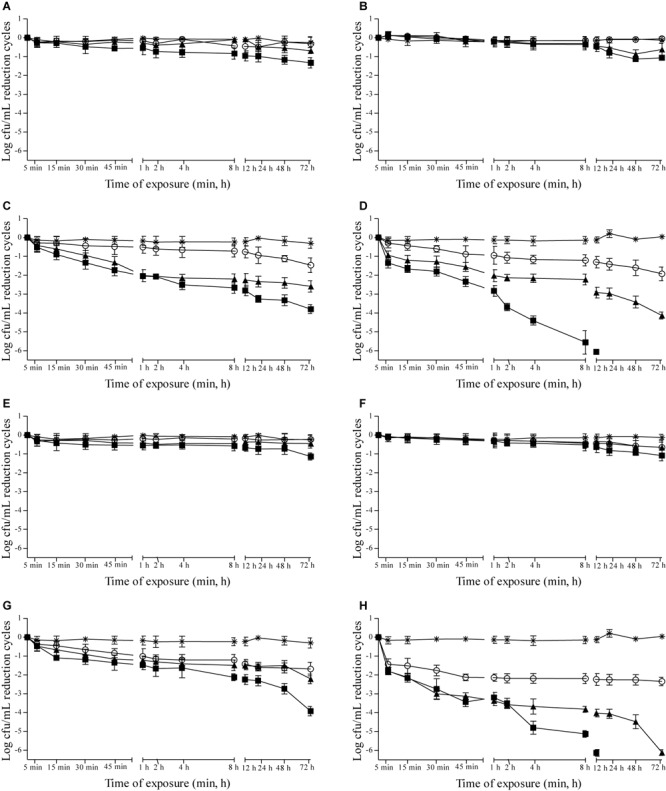
Reduction cycles (log cfu/mL; *n* = 6) of the counts of *C. albicans* ATCC 90028 **(A,E)**, *C. tropicalis* ATCC 28707 **(B,F)**, *P. anomala* ATCC 40101 **(C,G)** and *S. cerevisiae* ATCC 2601 **(D,H)** in pineapple juice at 4 ± 0.5°C as a function of the concentration of *M. spicata* L. essential oil **(A–D)** at (■): 3.75 μL/mL, (▲): 1.875 μL/mL, (∘): 0.9375 μL/mL or *M. villosa* Huds. essential oil **(E–H)**. (■): 15.0 μL/mL, (▲): 7.5 μL/mL, (∘): 3.75 μL/mL, (^∗^) control: 0 μL/mL. Detection limit of the test: 1 log cfu/mL.

Effects of 0.937, 1.875, and 3.75 μL/mL MSEO and 15, 7.5, and 3.75 μL/mL MVEO on the counts of *C. albicans, C. tropicalis, P. anomala*, and *S. cerevisiae* were studied in Sabouraud dextrose broth (Supplementary Figure S1) and in cashew (**Figure 1**), guava (**Figure 2**), mango (**Figure 3**), and pineapple (**Figure 4**) juices during 72 h of refrigerated storage. Incorporation of MSEO and MVEO at all assayed concentrations decreased (*p* ≤ 0.05) the counts of either of the tested yeast strains in Sabouraud dextrose broth as well as in fruit juices over time. Tested yeast strains presented a linear growth in Sabouraud dextrose broth and in fruit juices without MSEO and MVEO over the measured storage time period, with counts varying from 7 to 8 log cfu/mL.

Incorporation of 3.75 and 1.875 μL/mL MSEO in Sabouraud dextrose broth caused reductions in counts of *C. albicans*≥5-log and of 3.85-log cycles, respectively, after 72 h of exposure; the incorporation of 1.875 μL/mL MSEO in Sabouraud dextrose broth caused a ≥5-log reduction in counts of *P. anomala* after 72 h of exposure, while a higher concentration of 3.75 μL/mL was necessary to reduce ≥5-log in counts of *S. cerevisiae* after 24 h. In relation to MVEO, only the concentration of 15 μL/mL in Sabouraud dextrose broth reduced ≥5-log in counts of *C. albicans, P. anomala*, and *S. cerevisiae* after 72, 12, and 72 h of exposure, respectively (Supplementary Figures S1A–H).

In cashew juice, MSEO and MVEO were effective to cause ≥ 5-log reductions in counts of the tested yeast strains over the measured 72 h-storage period, with the exception of *C. tropicalis*. Incorporation of 3.75 and 1.875 μL/mL MSEO in cashew juice caused ≥5-log reductions in counts of *P. anomala* after 48 and 72 h of exposure, respectively (**Figure 1C**); the same reduction was observed in the counts of *S. cerevisiae* after an exposure time interval of 1 and 72 h, respectively (**Figure 1D**). Incorporation of 3.75 μL/mL MSEO or 15 μL/mL MVEO caused ≥5-log reductions in counts of *C. albicans* after 72 h of exposure (**Figures 1A,E**). Cashew juice containing 15 μL/mL MVEO presented ≥5-log reductions in counts of *P. anomala* and *S. cerevisiae* after 72 and 24 h of exposure, respectively (**Figures 1G,H**).

In guava juice, MSEO and MVEO were effective to cause ≥5-log reductions in counts of *C. albicans, P. anomala*, and *S. cerevisiae* over the monitored 72 h-storage period. Incorporation of 3.75 and 1.875 μL/mL MSEO in guava juice caused ≥5-log reduction in counts of *P. anomala* in an exposure time interval of 4 h (**Figure 2C**); 3.75 and 1.875 μL/mL MSEO caused ≥5-log reductions in counts of *S. cerevisiae* after 45 min and 8 h of exposure, respectively (**Figure 2D**); incorporation of 3.75 μL/mL MSEO or 15 μL/mL MVEO showed ≥5-log reductions in counts of *C. albicans* after 72 and 48 h of exposure, respectively (**Figures 2A,E**). Incorporation of 15 μL/mL MVEO in guava juice induced ≥5-log reductions in counts of *P. anomala* after 72 h of exposure (**Figure 2G**); this same reduction level was caused by 15 and 7.5 μL/mL MVEO against *S. cerevisiae* after 12 and 48 h of exposure, respectively (**Figure 2H**).

Incorporation of MSEO and MVEO at all tested concentrations was not effective to cause ≥5-log reductions in counts of *C. albicans, C. tropicalis*, and *P. anomala* in mango juice over the measured 72 h-storage period. Highest reductions in counts of *C. albicans* (1.33-log and 1.13-log), *C. tropicalis* (1.06-log and 1.08-log), and *P. anomala* (3.79-log and 3.92-log) at the end of the measured storage time period were observed in mango juice containing 3.75 μL/mL MSEO (**Figures 3A–C**) and 15 μL/mL MVEO (**Figures 3E–G**). Incorporation of 3.75 μL/mL MSEO or 15 μL/mL MVEO in mango juice caused ≥5-log reductions in counts of *S. cerevisiae* after 8 h of exposure (**Figures 3D,H**).

In pineapple juice with 3.75 μL/mL MSEO, ≥5-log reductions in counts of *P. anomala* and *S. cerevisiae* were observed after 48 h of exposure (**Figures 4C,D**). Only in pineapple juice containing 15 μL/mL MVEO a ≥5-log reduction in counts of *S. cerevisiae* was observed after a 4 h-exposure (**Figure 4H**). *C. albicans* and *C. tropicalis* showed count reductions of 1.63-log and 2.45-log cycles, respectively, in pineapple juice with 15 μL/mL MVEO after 72 h of exposure (**Figures 4E,F**).

Considering the data obtained in time-kill studies, the general ranking of sensitivity to MSEO and MVEO was *S. cerevisiae* > *P. anomala* > *C. albicans* > *C. tropicalis*; and the MSEO was more effective to cause ≥5-log reductions in yeasts counts than MVEO. Anti-yeast activity of MSEO and MVEO was dose-dependent, i.e., the reductions in yeasts counts were higher and occurred in a shorter exposure time when the concentrations of MSEO or MVEO increased. This dose-dependent effect was reported in other studies on the antimicrobial effects of *Mentha* essential oils (Tyagi et al., 2013; Guerra et al., 2015).

Results showing the ability of MSEO and MVEO to cause reductions in counts of tested yeasts in laboratorial media as well as in fruit juices are noteworthy because some previous studies have found that, in most cases, the antimicrobial efficacy of essential oils decreases when these substances are incorporated in food models (Gutierrez et al., 2009). Generally, MSEO and MVEO caused the lowest decreases in yeasts counts in mango juice that presents higher total soluble solids content than guava, pineapple, and cashew juices (de Sousa Guedes et al., 2016), reinforcing that carbohydrates may reduce the antimicrobial effects of essential oils (Gutierrez et al., 2009). Highest reductions in yeast counts caused by MSEO in Sabouraud dextrose broth as well as in fruit juices when compared with MVEO may be associated with the majority constituents found in each essential oil. Carvone (the majority constituent found in MSEO) typically presents stronger antifungal effects than piperitone oxide (the majority constituent found in MVEO). Higher solubility of carvone may also result in better capacity of the former to migrate across the characteristic aqueous medium of fruit juices and, consequently, to interact with yeast target cells to cause damage in cell structure and function (Trombetta et al., 2005; Soković et al., 2009).

There are no previous reports on the anti-yeast effects of MSEO or MVEO in fruit juices; however, previous studies with essential oils from other *Mentha* species have been reported. The detection of 2-log reductions in counts of *S. cerevisiae* in a mixed fruit juice (orange and apple) containing 1.13 mg/mL *M. piperita* essential oil was observed after 8 days of room temperature storage (Tyagi et al., 2013). Another study detected that the incorporation of 1 μL/mL *M. piperita* EO in apple juice caused ≥5-log reductions in counts of *Zygosaccharomyces rouxii* and *Z. bailii* after 15 days of refrigerated storage (Karaman et al., 2016).

The essential oil concentrations and the exposure time needed to achieve ≥5-log reductions in yeasts counts in cashew, guava, mango, or pineapple juices containing MSEO or MVEO observed in the present study were more than those previously observed for *E. coli, L. monocytogenes* and *Salmonella* Enteritidis in the same fruit juices containing *M. piperita* or *M. arvensis* essential oil (5, 2.5, and 1.25 μL/mL) (de Sousa Guedes et al., 2016). The needed essential oil concentration and the shorter time to achieve ≥5-log reductions in bacterial counts in juices containing *Mentha* essential oils could be associated with the higher sensitivity of bacterial cells to the acidic pH of fruit juices than yeast cells. Acidic pH in fruit juice may injury cells of low-pH sensitive microorganisms resulting in increased sensitivity to the action of essential oils constituents (Gutierrez et al., 2009; Tserennadmid et al., 2011).

Considering the results of assays that measured the anti-yeast effects of the tested essential oils, which demonstrated that MSEO even in lower doses than MVEO caused the highest reductions in yeasts counts in laboratorial media and in fruit juices, as well as the fact that high doses of *Mentha* essential oils typically impact negatively on the sensory attributes of fruit juices (de Sousa Guedes et al., 2016), only the MSEO in a effective anti-yeast concentration of 1.875 μL/mL was selected for use in physicochemical and sensory analyses of juices.

### Physicochemical and Sensory Characteristics of Fruit Juices

Considering that during the storage time fruit juice may present alterations in their physicochemical parameters impacting negatively on their quality aspects and market value, this study evaluated changes in selected physicochemical characteristics, namely °Brix, pH and titratable acidity, in cashew, guava, mango and pineapple juices with and without MSEO (1.875 μL/mL). These physicochemical characteristics were evaluated immediately after the essential oil addition and after 72 h of refrigerated storage (Supplementary Table S1). Overall, no difference (*p* > 0.05) in values of physicochemical parameters of juices with or without MSEO was observed, as well as at time zero and after 72 h of refrigerated storage. Juice samples with and without MSEO attended the criteria determined by the current Brazilian standard for unsweetened cashew, guava, mango, and pineapple juices, which determines titratable acidity (grams of citric acid per 100 g) values ≥0.15, ≥0.30, ≥0.30, and ≥0.16 and °Brix ≥ 5.0, ≥6.0, ≥10, and ≥6.0, respectively (Anonymous, 2003). These results are important because the physicochemical stability confirms that the studied fruit juices containing a effective anti-yeast dose of MSEO remain similar to those newly manufactured even after storage.

Results of the sensory analysis of cashew, guava, mango, and pineapple juice with 1.875 μL/mL MSEO are presented in **Table 3**. Considering the different sensory attribute terms established by panelists to describe the studied fruit juices in CATA test, some positive characteristics were observed in fruit juices with MSEO, such as the characteristic juice color and pleasant taste. Perception of the panelists indicated that most of the fruit juices tested presented high percent scores for non-characteristic fruit aroma, mint odor and taste and refreshing sensation. These sensory characteristics in juices could be associated with the presence of carvone, the majority constituent identified in the tested MSEO, which may impose these specific sensory characteristics in juices (Jakli and Saupe, 2006).

**Table 3:** Attribute terms percentage of the check-all-that-apply (CATA) questions of cashew, guava, mango, and pineapple juices with *Mentha spicata* L. essential oil (1.875 μL/mL).

Attribute terms	Juices			
	Cashew	Guava	Mango	Pineapple
	(%)	(%)	(%)	(%)
Characteristic color of the fruit juice	99^ab^	100^a^	98^ab^	96^b^
Uncharacteristic color of the fruit juice	1^ab^	0^b^	3^ab^	5^a^
Characteristic odor of the fruit juice	36^a^	19^b^	47^a^	14^b^
Uncharacteristic odor of the fruit juice	45^b^	57^a^	39^b^	64^a^
Mint odor	85^b^	88^ab^	73^c^	96^a^
Sweet	60^a^	34^b^	54^a^	42^b^
Not sweet	27^b^	55^a^	32^bc^	42^c^
Strange flavor	20^b^	35^a^	21^b^	25^b^
Refreshing sensation	89^a^	86^a^	60^b^	84^a^
Fruit flavor	42^a^	29^b^	19^c^	22^bc^
Mint flavor	88^a^	87^a^	69^b^	88^a^
Bitter flavor	13^bc^	27^a^	18^b^	7^c^
Pleasant flavor	67^a^	37^c^	52^b^	54^b^
Unpleasant flavor	9^b^	19^a^	16^a^	19^a^

*^*a*-*c*^Different superscript letters within a row are significantly different (*p* ≤ 0.05), based on Cochran’s Q test.*

Overall, the results of CATA test showed that the incorporation of 1.875 μL/mL MSEO in cashew, guava, mango, and pineapple juice did not affect negatively their sensory characteristics enough to imply on potential rejection by consumers. These results are noteworthy because a previous study observed that the incorporation of an effective antibacterial dose (1.25 μL) of *M. piperita* essential oil in cashew, mango, pineapple, and guava juice affected negatively the taste, aftertaste and overall acceptance of juices, although no negative effects have been observed in appearance, flavor and odor (de Sousa Guedes et al., 2016).

## Conclusion

Results of this study showed that MSEO (3.75, 1.875, and 0.937 μL/mL) or MVEO (15.0, 7.5, and 3.75 μL/mL) are capable to inactivate *C. albicans, C. tropicalis, P. anomala*, and *S. cerevisiae* in laboratorial media and in cashew, guava, mango and pineapple juice over a 72 h-refrigerated storage. Overall, MSEO showed stronger anti-yeast effects than MVEO; *S. cerevisiae* and *C. tropicalis* were the most and less sensitive yeasts, respectively, to both studied essential oils; and the highest yeast count reductions were verified in cashew and guava juice. An effective anti-yeast dose of MSEO (1.875 μL/mL) did not affect the physicochemical parameters comprising the identity and quality characteristics for unsweetened juices, as well as did not induce negative alterations to cause possible sensory rejection of tested fruit juices. These results indicate MSEO and MVEO, primarily MSEO, as potential antimicrobials to comprise preservation strategies used to control spoilage yeasts in fruit juices.

## Author Contributions

ES, EC, and MM: conceived and designed the experiments and drafted the paper. EC, IM, ES, MM, JB-F, and JT: performed the experiments. EC, IM, ES, MM, and JT: analyzed the data.

## Conflict of Interest Statement

The authors declare that the research was conducted in the absence of any commercial or financial relationships that could be construed as a potential conflict of interest.
