# Supplementary material for: Inactivation of Spoilage Yeasts by Mentha spicata L. and M. × villosa Huds. Essential Oils in Cashew, Guava, Mango, and Pineapple Juices
Source: Front Microbiol. 2018 May 25;9:1111. doi: 10.3389/fmicb.2018.01111 (PMC5981176; doi:10.3389/fmicb.2018.01111)
Supplement: Supplementary file 1 [file Data_Sheet_1.pdf]

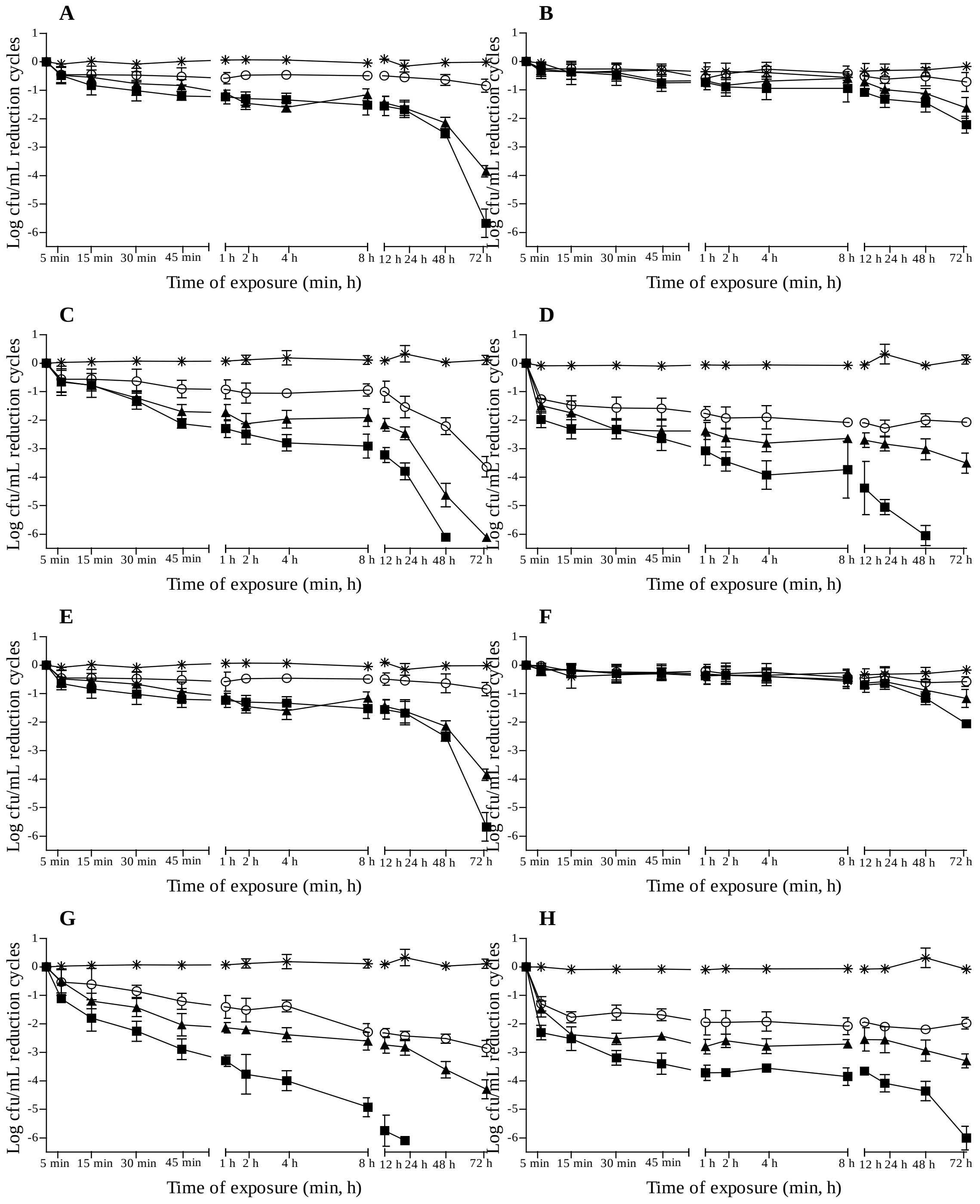

Supplementary Figure S1. Reduction cycles (log cfu/mL) of the counts of *C. albicans* ATCC 90028 (A, E), *C. tropicalis* ATCC 28707 (B, F), *P. anomala* ATCC 40101 (C, G) and *S. cerevisiae* ATCC 2601 (D, H) in sabouraud dextrose broth at 4 ± 0.5 ºC as a function of the concentration of *M. spicata* L. essential oil (A – D) at (■): 3.75 µL/mL, (▲): 1.875 µL/mL, (○): 0.9375 µL/mL or *M. villosa* Huds. essential oil (E – H). (■): 15.0 µL/mL, (▲): 7.5 µL/mL, (○): 3.75 µL/mL, (∗) control: 0 µL/mL. Detection limit of the test: 1 log cfu/mL.

Supplementary Table S1. Physicochemical parameters (average ± standard deviation; n = 6) of cashew, guava, mango and pineapple juices with and without 1.875 µL/mL of *Mentha spicata* L. essential oil (MSEO) immediately after the essential oil incorporation (time zero) and after 72 h of refrigerated storage (4 ± 0.5 ºC).

Juices	Treatments	Physicochemical parameters (storage time interval)					
		Total soluble solids (°Brix)		pH		Titratable acidity (g/100 g citric acid)	
		Time zero	72 h	Time zero	72 h	Time zero	72 h
Cashew	MSEO (1.875 µL/mL)	5.70 (±0.30)^{Aa}	5.80 (±0.20)^{Aa}	4.86 (±0.15)^{Aa}	4.89 (±0.23)^{Aa}	0.17 (±0.08)^{Aa}	0.18 (±0.02)^{Aa}
	Control (0 µL/mL)	5.60 (±0.20)^{Aa}	5.70 (±0.30)^{Aa}	4.88 (±0.32)^{Aa}	4.84 (±0.20)^{Aa}	0.17 (±0.03)^{Aa}	0.19 (±0.02)^{Aa}
Guava	MSEO (1.875 µL/mL)	6.10 (±0.30)^{Aa}	6.10 (±0.20)^{Aa}	4.08 (±0.35)^{Aa}	4.12 (±0.25)^{Aa}	0.34 (±0.06)^{Aa}	0.33 (±0.03)^{Aa}
	Control (0 µL/mL)	6.20 (±0.30)^{Aa}	6.30 (±0.20)^{Aa}	4.14 (±0.45)^{Aa}	4.12 (±0.21)^{Aa}	0.35 (±0.08)^{Aa}	0.34 (±0.02)^{Aa}
Mango	MSEO (1.875 µL/mL)	10.20 (±0.10)^{Aa}	10.30 (±0.10)^{Aa}	44.74 (±0.25)^{Aa}	44.69 (±0.25)^{Aa}	0.32 (±0.06)^{Aa}	0.32 (±0.02)^{Aa}
	Control (0 µL/mL)	10.20 (±0.20)^{Aa}	10.30 (±0.20)^{Aa}	44.75 (±0.17)^{Aa}	44.71 (±0.24)^{Aa}	0.32 (±0.07)^{Aa}	0.34 (±0.02)^{Aa}
Pineapple	MSEO (1.875 µL/mL)	6.60 (±0.30)^{Aa}	6.60 (±0.20)^{Aa}	4.63 (±0.24)^{Aa}	4.58 (±0.20)^{Aa}	0.23 (±0.03)^{Aa}	0.22 (±0.02)^{Aa}
	Control (0 µL/mL)	6.70 (±0.20)^{Aa}	6.60 (±0.20)^{Aa}	4.65 (±0.14)^{Aa}	4.59 (±0.30)^{Aa}	0.24 (±0.02)^{Aa}	0.24 (±0.01)^{Aa}

Control (0 µL/mL): fruit juice without the incorporation of MSEO.
Similar superscript capital letters in the same row for the same fruit juice and essential oil concentration indicate no significant difference (p > 0.05), based on student t test.
Similar superscript small letters in the same column for the same fruit juice indicate no significant difference (p > 0.05), based on student t test.
